# Disabled Warriors and Parliament

**Published:** 1915-09-11

**Authors:** 


### DISABLED WARRIORS AND PARLIAMENT.

### Their First Claim on the Nation's Resources.

England has been far behind other nations in the care and provision she has heretofore made for wounded and permanently disabled sailors and soldiers in the conduct of wars waged by the nation. It has long been, and is even still, a national disgrace that old warriors are to be found in workhouses, and that some of the most valiant of the rank and file who have received recognition for bravery and service should in their old age to-day be left by the nation without adequate provision. For many years we have, in season and out of season, dealt with this question, brought out the cruelties which have attached to past neglect, and pleaded diligently for the application of an adequate remedy.

When the great meeting was held at the Guildhall, at which Mr. Asquith and Mr. Bonar Law spoke, the latter described the warriors we refer to as children of the State who should be cared for as a sacred trust by the nation. So ingrained, however, in those who have been mainly responsible for the War Office and local government in this country are ancient and evil systems, that on the establishment of the Prince of Wales's National Relief Fund one of the early steps was to approve a scheme by which the wives and dependants of our warriors at the Front should obtain their allowances by application at the offices of the Board of Guardians. We believe that the sanction of the Local Government Board to this arrangement was actually obtained. Fancy handing over the dependants of our sailors and soldiers to the administrators of the Poor Law, to the managers of charities, and to Distress Committees! Our readers will remember that we fought this question strongly, and exposed its wanton cruelty and injustice in a leading article which appeared in The Hospital of September 19, 1914. In the result, despite the opposition of Mr. Hobhouse, the then Postmaster-General, and his Department, the Government was induced to adopt the plan we advocated—*i.e.* to pay the wives and dependants weekly through the Post Office. In this way the Post Office has become the bank of the dependants of our warriors, every one of whom has a cheque-book and attends every week at the nearest post office, where the cheque-book is kept, to draw a cheque for the amount to which each of them may be entitled. This simple plan has preserved the independence and self-respect of the dear ones of our sailors and soldiers who are fighting the nation's battle, and is calculated to exercise a material influence in securing a continuous and proper maintenance of each home during the absence of the warrior at the Front.

The Pensions Bill properly provides for the setting up of an independent organisation under Government control, through which all the business relating to pensions for our warriors and those dependent upon them shall be conducted in future. This plan is worthy of the nation, for the time has come to put this matter on a national and adequate basis once and for all in justice to our sailors and soldiers, who are children of the State and have the first claim upon the resources of the nation. Unfortunately the House of Lords has hung up the Pensions Bill because, as we cannot but believe through some misapprehension, a small active opposition was got up in that House with the object, as we understand the matter, to secure that the pension provision for our sailors and soldiers shall have about it several of the old charity and philanthropic evils. We are fully conscious of the good and bad points of the work done in the past through private benevolence. But the decision of the Government to ensure permanence in the future in the arrangements for the provision and distribution of grants in this connection is wise, for it is the only possible way to end for ever the evils, abuses, and injustices of the past. Everything that is good and worthy of utilisation under the old plan will no doubt be weighed and made use of to the fullest extent desirable. We are all grateful for the services rendered by private benevolence and those associated with it in the past, but the great war necessitates that our sailors and soldiers shall from henceforward be treated as children of the State, and that their claims shall be met out of the resources of the nation quite independently of every taint of charity and of the evils which have attached to that word and its application in regard to our sailors and soldiers in past decades. We hope, therefore, that the Pensions Bill, which sets up an entirely new and independent authority with complete freedom to create and perfect an organisation which for all time shall adequately provide for and protect our warriors of all ranks who may be injured by war, their wives, children, and dependants, will receive the approval of Parliament and be converted into an Act immediately after its re-assembling.

### Sir F. Treves' Tribute.

British people of all ranks have far too dim an apprehension of the miseries and sufferings of our warriors who are permanently disabled by war. This ignorance should be lessened by Her Majesty the Queen's acceptance of the gift of the Star and Garter Hotel at Richmond; it will be devoted to the care of such totally disabled men as may be retired from the Services. With the same end in view we have great pleasure in reproducing an able monograph by Sir Frederick Treves, which sets forth the position and needs of our permanently injured warriors forcibly and well:—

"In the aftermath of this grievous war there will be no more lamentable and pathetic figure than the soldier who, by reason of his wounds, is paralysed and left utterly helpless. One is apt to associate such helplessness with extreme old age or with the final phase of some exhausting illness; but here is a man in the very flower of his youth, bedridden for life, unable to move hand or foot, and dependent, at every moment of the day, upon the ministration of others. In the paralysis of old age there is usually, coincident with the loss of power, a merciful decay of the brain, a loss of mind that merges into mere apathy and oblivion. In the case of the stricken soldier, however, the mind is as vigorous and as alert as ever; the eagerness and independence of youth are still aglow in the brain; there are still the intense longing to do, the stimulus to venture, the desire to lay hold of the joys of life; while with this mental energy is associated a body that cannot feel, limbs that cannot move, fingers without touch, and hands as listless as the hands of the dead. Indeed, there is no more piteous spectacle of human wreckage than this nerveless body, abject in its impotence and decrepitude, linked with the restless and imperious spirit of sturdy manhood; in the headlong rush of the bayonet charge there has been a sudden stab of pain, and the man of arms, terrible in his strength, has become in a moment feebler than a child. He has lost everything but bare life, and, saddest of all, there is taken from him the dearest of all treasures —Hope. Let the people of this country who are safe and sound remember that it was in fighting for England that such dire ruin fell upon these men, and that it rests with those for whom they fought to afford them whatever comfort it is within the power of man to bestow. After every form of treatment has been attempted and failed, the soldier who is the subject of severe paralysis is retired from the Army with a small pension. It is generally assumed that he will return to his friends to end his days, and that his pension will defray the cost of his maintenance and care. In the average case this is an impossible position. That he will be treated in his home with affectionate devotion none will doubt, but in cases such as these something more than devotion is needed, if the patient's life is to be prolonged and if he is to be spared from suffering while life lasts. He will need skilled and indeed special nursing. The cottage bedroom is often small and cramped, and in every way unsuited for the care of cases of this type. The possibility of taking advantage of any but the simplest measure of treatment is slight, while the prospect of a bed being moved day by day into the open air is very remote. When the difficulties and disadvantages are grasped and found insuperable, one knows what happens—the patient is moved to the wards of a workhouse infirmary, and there his career comes to an end with little glory to those who say that the wounded soldier shall lack for nothing and that England is grateful to all who have fought for her freedom. It rests with the well-to-do to rid the country of reproach in this matter, and to perform not merely an act of kindness, but an act of justice. This Hostel and Garden City will be the last home of the disabled soldier. It can be made worthy of him if the people of England will make it worthy of the country for which he has suffered. He has fought his fight; he has met his fate; let him feel that the loving kindness of the land for which he has given his life is around him when the bugle from afar off summons him to ' The Last Post.' "

